# Comparison of Myocardial Injury and Inflammation Biomarkers and Their Impact on Recurrence after Cryoballoon and Radiofrequency Ablation for Atrial Fibrillation: A Systematic Review and Meta-Analysis

**DOI:** 10.31083/j.rcm2312397

**Published:** 2022-12-07

**Authors:** Xinbin Zhou, Hong Xue, Qian Chen, Zhengtian Lv, Wei Mao, Xiao Wang

**Affiliations:** ^1^Department of Cardiology, The First Affiliated Hospital of Zhejiang Chinese Medical University (Zhejiang Provincial Hospital of Chinese Medicine), Key Laboratory of Integrative Chinese and Western Medicine for the Diagnosis and Treatment of Circulatory Diseases of Zhejiang Province, 310006 Hangzhou, Zhejiang, China; ^2^Department of Cardiology, Qingdao Hospital of Traditional Chinese Medicine (Qingdao Hiser Hospital), 266000 Qingdao, Shandong, China; ^3^The First College of Clinical Medicine, Zhejiang Chinese Medical University, 310006 Hangzhou, Zhejiang, China; ^4^Department of Geriatrics, The First Affiliated Hospital of Zhejiang Chinese Medical University (Zhejiang Provincial Hospital of Chinese Medicine), 310006 Hangzhou, Zhejiang, China

**Keywords:** atrial fibrillation, cryoballoon ablation, radiofrequency ablation, myocardial injury biomarkers, inflammation biomarkers, meta-analysis

## Abstract

**Background:**

Biomarkers of myocardial injury and inflammation were found to be different after radiofrequency catheter ablation (RFCA) and cryoballoon ablation (CBA) for atrial fibrillation (AF); however, the results are currently controversial. This study was aimed to systematically compare the differences in myocardial injury and inflammation biomarkers after RFCA and CBA procedures and to investigate their impact on AF recurrence.

**Methods:**

Databases, including PubMed, Embase, the Cochrane Library, ClinicalTrials.gov, China National Knowledge Infrastructure (CNKI) and China Biology Medicine (CBM), were systematically searched from their date of inception to May 2022. The primary outcomes of interest were the differences in myocardial injury and inflammation biomarkers after CBA and RFCA procedures for AF patients, and the impact of the biomarkers on AF recurrence. Secondary outcomes included the total ablation time, the procedure duration and the freedom from atrial tachycardia (AT).

**Results:**

Eighteen studies with a total of 1807 patients were finally enrolled. CBA treatment was associated with significantly greater increases in troponin I (TNI) levels (weighted mean difference [WMD] = 3.13 ug/L, 95% confidence interval [CI] 2.43–3.64) both at 4–6 h (WMD = 3.94 ug/L), 24 h (WMD = 4.23 ug/L), 48 h (WMD = 2.14 ug/L) and 72 h (WMD = 0.56 ug/L), and also creatine kinade MB fraction (CK-MB) levels at 4–6 h (WMD = 33.21 U/L), 24 h (WMD = 35.84 U/L) and 48 h (WMD = 4.62 U/L), while RFCA treatment was associated with greater increases in postablation C-reactive protein (CRP) levels both at 48 h (WMD = –9.32 mg/L) and 72 h (WMD = –10.90 mg/L). The CBA and RFCA treatments had comparable rates of freedom from AT (74.5% vs. 75.2%, RR = 1.08). The CRP levels were significantly higher in patients with early recurrence of AF (ERAF) than in those without ERAF after RFCA treatment (WMD = 3.415 mg/L).

**Conclusions:**

The time-course patterns of postablation myocardial injury and inflammation biomarkers are different between RFCA and CBA procedures. The lower postprocedural elevation of myocardial injury biomarkers and the increased CRP levels may be predictive factors for ERAF.

**PROSPERO Registration Number:**

CRD42021278564.

## 1. Introduction

Atrial fibrillation (AF) is the most common cardiac arrhythmia and is associated with a significantly increased risk of morbidity and mortality [1, 2]. For symptomatic and drug-refractory AF patients, pulmonary vein isolation (PVI) by catheter ablation (CA) has been the cornerstone therapeutic option to restore and maintain sinus rhythm and to improve the quality of life [2]. Point-by-point radiofrequency catheter ablation (RFCA) and balloon-based cryoballoon ablation (CBA) are the two widespread ablation procedures, with comparable efficacy and safety [3, 4, 5].

Previous studies demonstrated that various biomarkers of myocardial injury and inflammation, including troponin I (TNI), troponin T (TNT), creatine kinase (CK), creatine kinase MB fraction (CK-MB) and C-reactive protein (CRP), are elevated in patients undergoing RFCA and CBA for AF [6, 7, 8]. The characteristics of myocardial injury and inflammation biomarkers may differ between RFCA and CBA, due to their considerable differences in lesion formation [8, 9]. The lesions created by RFCA have ragged boundaries with more extensive endothelial disruption, while those created by CBA have well-circumscribed, discrete lesions, and the tissue ultrastructure is preserved [10, 11, 12, 13].

The differences and patterns of myocardial injury and inflammation biomarkers have been investigated in many published studies; however, the results are currently controversial [14, 15, 16, 17, 18]. In addition, the relationships between these biomarkers after the CA procedure and clinical outcomes, including early/late recurrences of AF, remain unclear [8]. In this study, we aimed to analyze and compare the patterns of myocardial injury and inflammation biomarkers after RFCA and CBA procedures for AF patients, and the relationships between these markers and the recurrence of AF were further evaluated.

## 2. Materials and Methods

### 2.1 Search Strategy and Selection Criteria

PubMed, Embase, the Cochrane Library, ClinicalTrials.gov and the databases of the China National Knowledge Infrastructure (CNKI), Wanfang, and China Biology Medicine (CBM) were systematically searched up to May, 2022. The following terms and variants thereof were used: “myocardial injury biomarker”, “cryoballoon”, “radiofrequency”, and “atrial fibrillation”. In addition, the references of the selected articles and relevant reviews were manually searched for potentially relevant studies. Only full-text articles that reported outcomes of interest were included; no language restriction was imposed.

### 2.2 Data Collection and Quality Assessment

Data extraction and quality assessment were performed by two investigators independently and the discrepancies were resolved by consensus. The following data were extracted: patient number, participant characteristics, ablation strategy, duration of follow-up and outcomes of interest. The quality of the included randomized controlled trials (RCTs) was assessed with the Cochrane Collaboration tool [19], while the quality of the nonrandomized studies was evaluated using the ROBINS-I tool [20].

### 2.3 Primary and Secondary Outcomes 

The primary outcomes of interest were the differences in myocardial injury and inflammation biomarkers, including TNI levels (ug/L), TNT levels (ng/mL), CK-MB levels (U/L), CK levels (U/L) and CRP levels (mg/L), at different time points after the ablation procedure between CBA and RFCA therapy for AF patients. The impact of the abovementioned biomarkers on AF recurrence, including early recurrence of AF (ERAF) and late recurrence of AF (LRAF), was also investigated. ERAF was defined as atrial tachycardia (AT) including AF, atrial flutter or atrial tachycardia documented on the electrocardiogram (ECG) or Holter continuing longer than 30 seconds within 3 months after the CA procedure, while LRAF was defined as recurrence documented from 3 months to 1 year. Secondary outcomes included the total ablation time, procedure duration and freedom from AT during follow-up.

### 2.4 Statistical Analysis

Continuous variables were described as median and standard deviation (SD), and categorical variables were described as n (%). STATA version 12.0 (STATA Corporation, College Station, TX, USA) was applied to perform meta-analysis. Odds ratio (OR) and weighted mean difference (WMD) and the 95% confidence interval (CI) were calculated to demonstrate the overall result. Heterogeneity across studies was assessed with the chi-square test, and $I^{2}$ > 50% was considered indicative of significant heterogeneity. When significant heterogeneity was present, the possible causes were investigated. The publication bias was analyzed by funnel plots graphically and by Egger’s and Begg’s tests statistically. The protocol for this systematic review was registered on PROSPERO (doi: 10.15124/ CRD42021278564).

## 3. Results

### 3.1 Eligible Studies and Characteristics

**Fig. 1.**
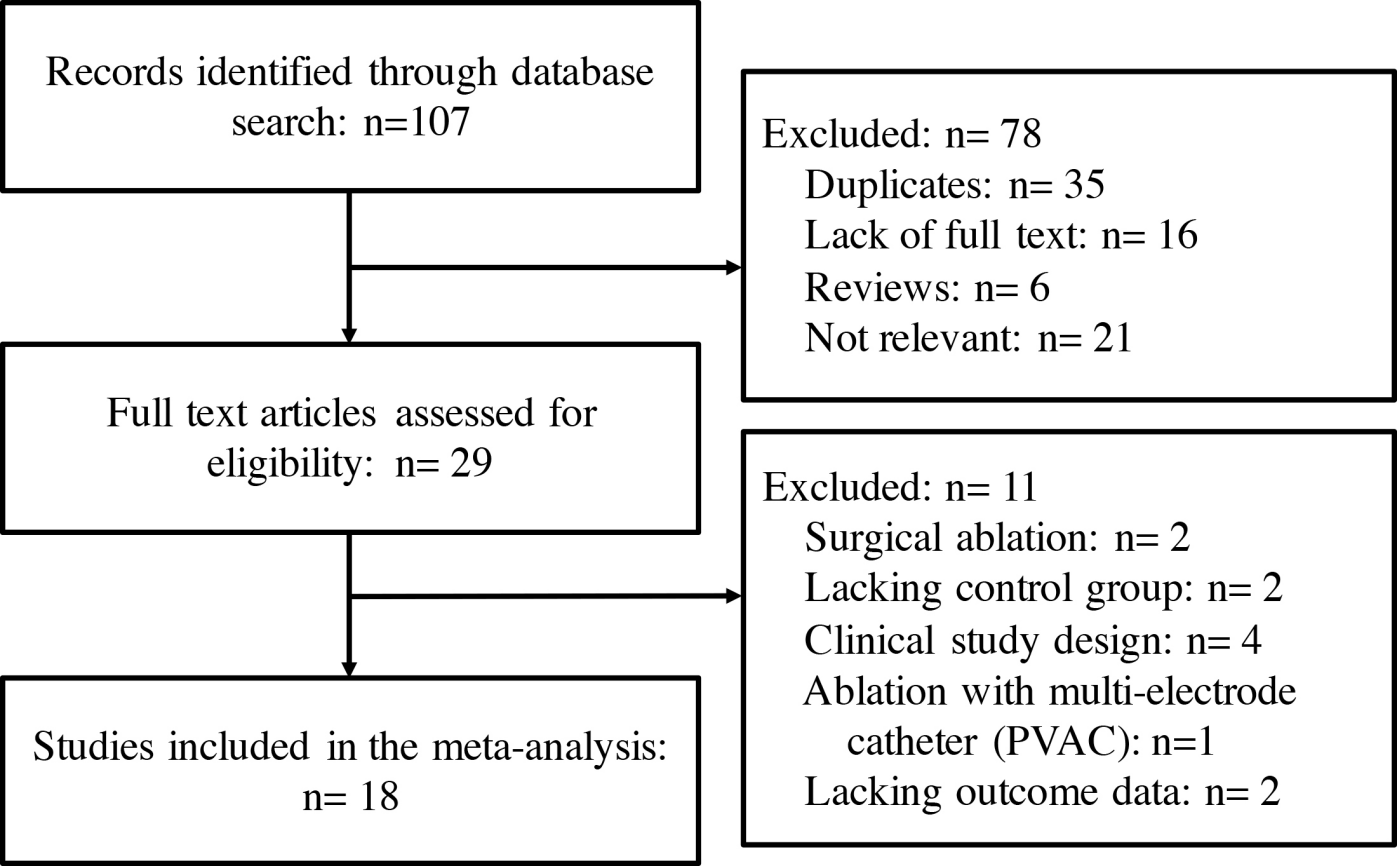
**Flow chart of the systematic literature research**.

**Fig. 2.**
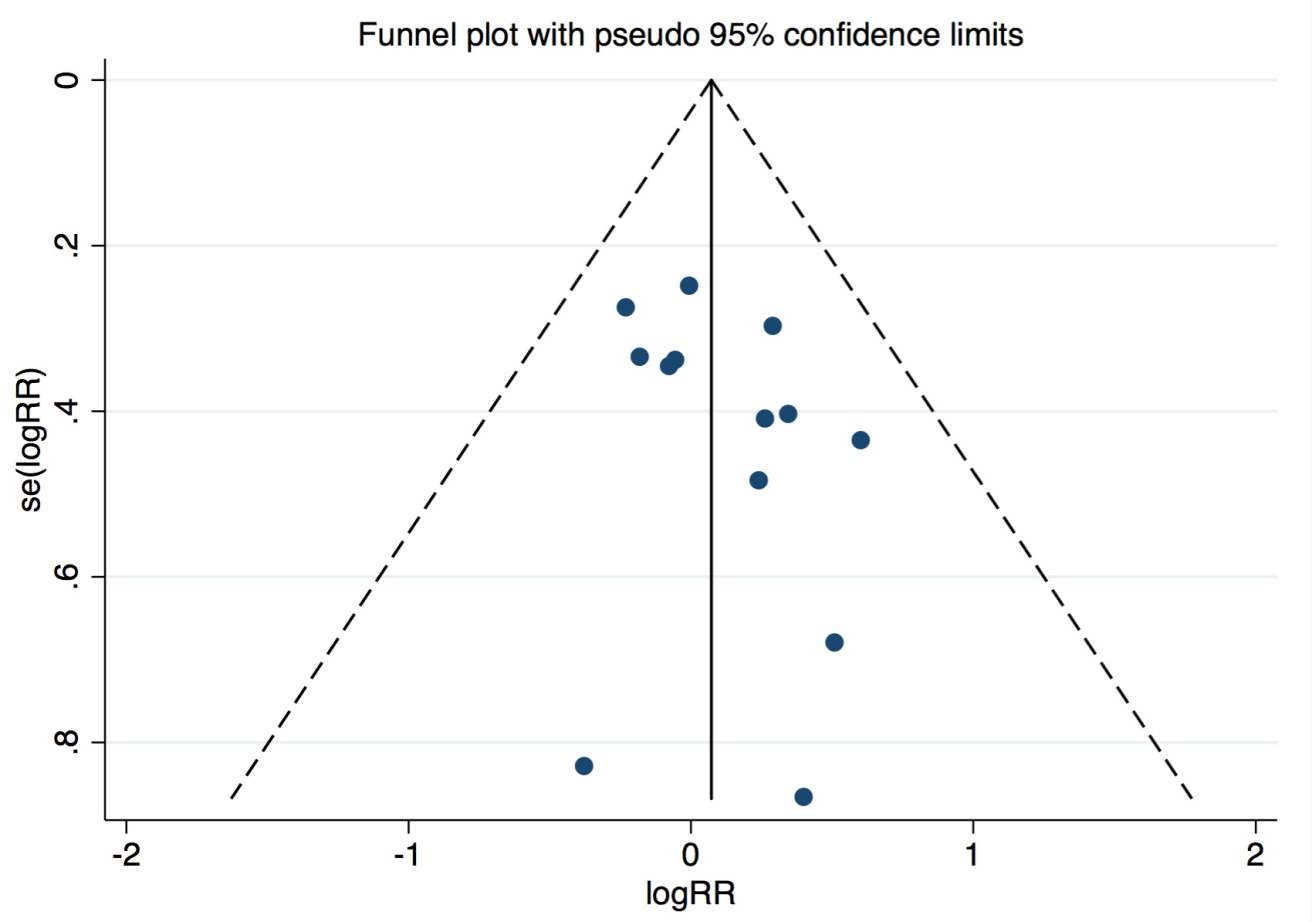
**Funnel plot for the studies included**.

A total of 107 potentially relevant studies were identified in the initial search, of which 29 studies were further assessed. Finally, 18 clinical trials [8, 14, 15, 16, 17, 21, 22, 23, 24, 25, 26, 27, 28, 29, 30, 31, 32, 33] with a total of 1807 patients were included in the meta-analysis (Fig. 1). No additional studies were identified. The baseline characteristics of the included studies are presented in Table 1 (Ref. [8, 14, 15, 16, 17, 21, 22, 23, 24, 25, 26, 27, 28, 29, 30, 31, 32, 33]). Briefly, across the trials, three studies [14, 15, 31] were RCTs, while the remaining studies were nonrandomized trials. Twelve studies [8, 14, 16, 17, 23, 24, 25, 28, 29, 30, 31, 33] included only paroxysmal AF (PAF) patients, and the remaining 6 studies [15, 21, 22, 26, 27, 32] included both PAF and persistent AF (PerAF) patients. In total, 852 patients were in the CBA group and 955 patients were in the RFCA group. The mean age of the patients ranged from 54.5 to 68.7 years. The mean left ventricular ejection fraction (LVEF) ranged from 47.1% to 68.3%, and the mean left atrium diameter (LAd) ranged from 35.5 mm to 43.8 mm. CBA with the second-generation CB (CB2) was applied in 11 studies [8, 16, 24, 25, 27, 28, 29, 30, 31, 32, 33], while CBA with the first-generation CB (CB1) or both CB1 and CB2 was applied in the remaining studies. RFCA with contact force technology (RF-CF) was applied in 7 studies [8, 16, 29, 30, 31, 32, 33], while RFCA without contact force technology (RF-nCF) was applied in 7 studies [15, 17, 21, 22, 23, 24, 26]. All included studies were of good quality according to the Cochrane Collaboration tool [19] and ROBINS-I tool [20]. No significant publication bias was found by funnel plot or Egger’s and Begg’s tests based on the primary outcomes (Egger’s: *p* = 0.203; Begg’s: *p* = 0.393) (Fig. 2).

**Table 1.** **Baseline characteristics of the included studies**.

References	Year	N	Patients (n)		Mean age (years)	Male, %	Mean LVEF (%)	Mean Lad (mm)	DM (%)	Hypertension (%)	CAD (%)	CBA protocol	RFCA protocol	Follow-up
			PAF	PerAF										
Yano *et al*. [8]	2020	263	263	0	68.6	58.6	68.3	42.5	15.2	61.2	NR	CB2	RF-CF	3 m
Minamisaka *et al*. [33]	2020	138	138	0	67.5	59.4	65.5	39	15.5	68.1	NR	CB2	RF-CF	12 m
Hisazaki *et al*. [16]	2019	86	86	0	65.5	64	67.5	35.5	NR	50	NR	CB2	RF-CF	20 m
You *et al*. [31]	2019	140	140	0	58.6	57.9	50.8	35.7	15.7	60.7	NR	CB2	RF-CF	12 m
Giannopoulos *et al*. [30]	2019	120	120	0	59.5	NR	60	40.8	12.5	49.2	6.7	CB2	RF-CF	6 m
Bin Waleed *et al*. [29]	2019	58	58	0	61.8	58.6	59	36.2	8.6	46.6	NR	CB2	RF-CF	6 m
Zeljkovic *et al*. [32]	2019	79	54	25	61.5	79.7	59.6	40	NR	50.6	NR	CB2	RF-CF	12 m
Kizilirmak *et al*. [26]	2017	98	76	22	54.5	51	63.5	39	15.3	51	14.3	CB1/2	RF-nCF	9 m
Yang *et al*. [27]	2018	45	38	7	62.5	62.2	66	41.6	11.1	57.8	8.9	CB2	NR	5 d
Wang *et al*. [28]	2018	102	102	0	61.5	65.7	63.5	40	9.8	54.9	NR	CB2	NR	3 m
Xiao *et al*. [25]	2016	80	80	0	68.7	47.5	47.1	43.8	NR	NR	12.5	CB2	NR	1 d
Antolic *et al*. [23]	2016	41	41	0	61	68.3	NR	NR	2.4	56.1	NR	CB1	RF-nCF	23 m
Miyazaki *et al*. [24]	2016	82	82	0	62.6	70.7	65.7	38.3	NR	51.2	NR	CB2	RF-nCF	3 m
Lian *et al*. [22]	2015	60	45	15	60.7	61.7	NR	NR	NR	NR	NR	CB1	RF-nCF	3 d
Casella *et al*. [14]	2014	90	90	0	57.3	74.4	62	42.2	NR	35.6	NR	CB1/2	RF-CF/nCF	12 m
Herrera Siklódy *et al*. [15]	2012	60	38	22	56.5	80	NR	40.7	NR	45	11.7	CB1	RF-nCF	24 m
Schmidt *et al*. [21]	2012	215	133	82	61.5	82.8	59	46	10.2	60	19.5	CB1	RF-nCF	24 h
Kühne *et al*. [17]	2010	50	50	0	58.5	86	59	41.5	NR	14	16	CB1	RF-nCF	12 m

PAF, paroxysmal atrial fibrillation; PerAF, persistent atrial fibrillation; LVEF, left ventricular ejection fraction; LAd, left atrial diameter; DM, diabetes mellitus; CAD, coronary artery disease; NR, not reported; CBA, cryoballoon ablation; RFCA, radiofrequency catheter ablation; CB1, CBA with the first-generation cryoballoon; CB2, CBA with the second-generation cryoballoon; RF-CF, RFCA with contact force technology; RF-nCF, RFCA without contact force technology.

### 3.2 Primary Endpoints

#### 3.2.1 TNI Levels

**Fig. 3.**
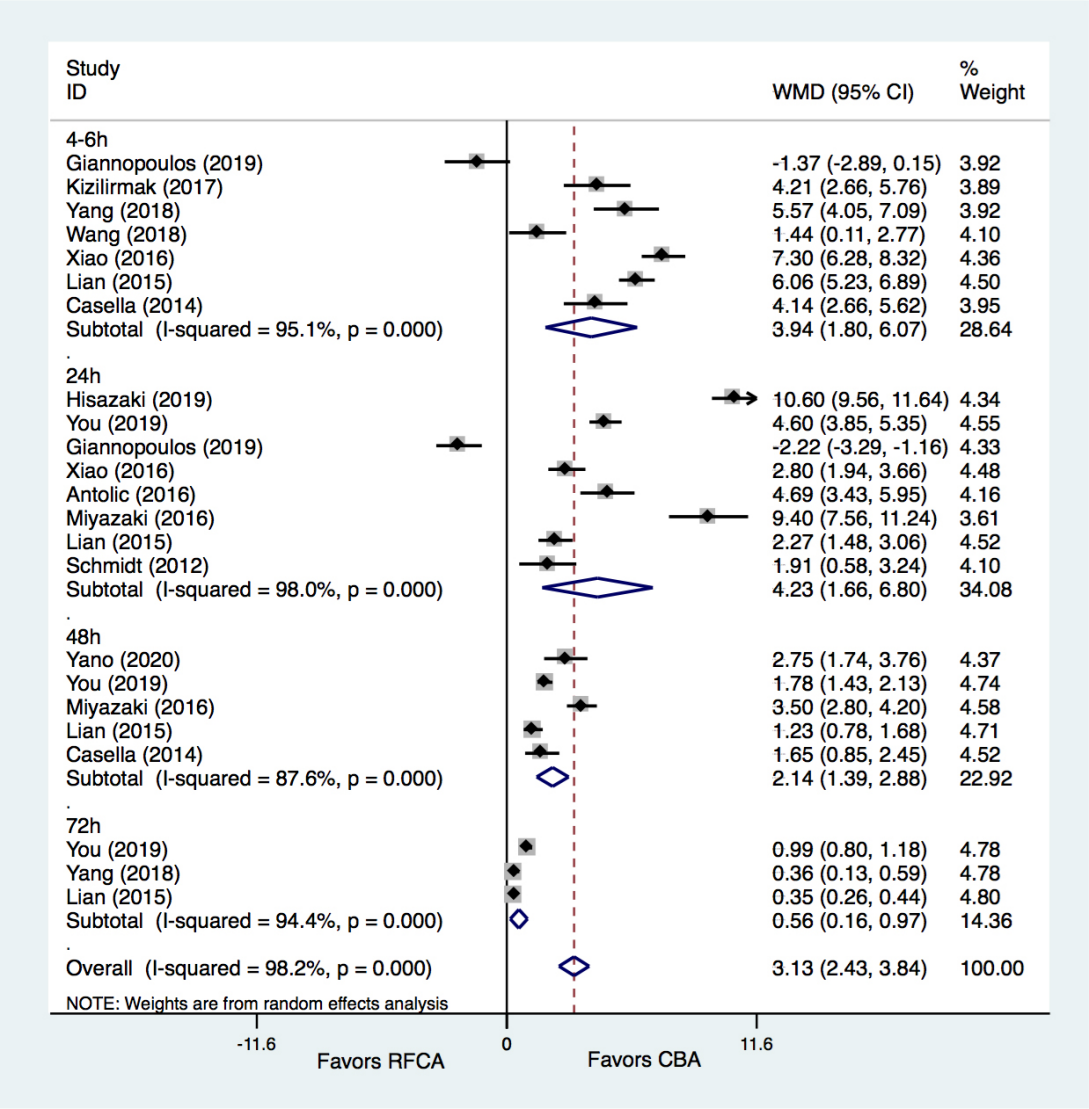
**Meta-analysis for the outcome of TNI**. RFCA, radiofrequency catheter ablation; CBA, cryoballoon ablation; WMD, weight mean difference.

Of the included trials, 13 studies [8, 14, 16, 21, 22, 23, 24, 25, 26, 27, 28, 30, 31] provided information on TNI levels at different time points after CBA or RFCA treatments. The results demonstrated that CBA treatment was associated with a significantly greater increase in TNI levels than RFCA treatment (WMD = 3.13 ug/L, 95% CI 2.43–3.64, *p* = 0.000). Compared with the RFCA group, the TNI levels increased greatly both at 4–6 h (WMD = 3.94 ug/L, 95% CI 1.80–6.07, *p* = 0.000), 24 h (WMD = 4.23 ug/L, 95% CI 1.66–6.80, *p* = 0.001), 48 h (WMD = 2.14 ug/L, 95% CI 1.39–2.88, *p* = 0.000) and also 72 h (WMD = 0.56 ug/L, 95% CI 0.16–0.97, *p* = 0.006) in the CBA group. However, moderate to significant heterogeneities were detected for the comparisons of TNI levels at different time points (Fig. 3).

#### 3.2.2 TNT Levels

**Fig. 4.**
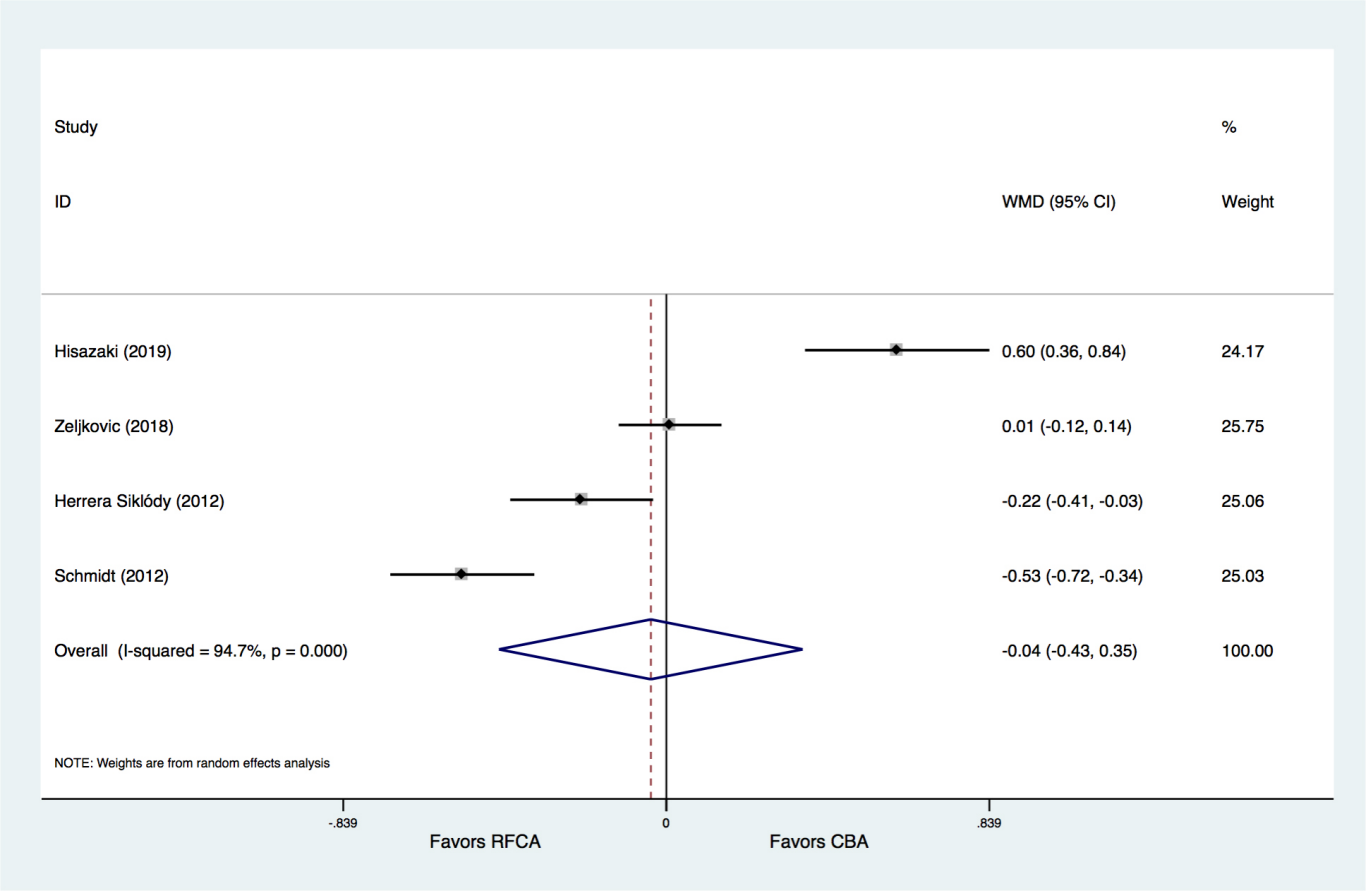
**Meta-analysis for the outcome of TNT**. RFCA, radiofrequency catheter ablation; CBA, cryoballoon ablation; WMD, weight mean difference.

Of the included trials, 4 studies [15, 16, 21, 32] provided information on TNT levels at 18–24 h after CBA or RFCA treatments. The results demonstrated that the TNT levels at 18–24 h did not significantly differ between the CBA and RFCA groups (WMD = –0.04 ng/mL, 95% CI –0.43–0.35, *p* = 0.842). Significant heterogeneity was observed for this endpoint (Fig. 4).

#### 3.2.3 CK-MB Levels

**Fig. 5.**
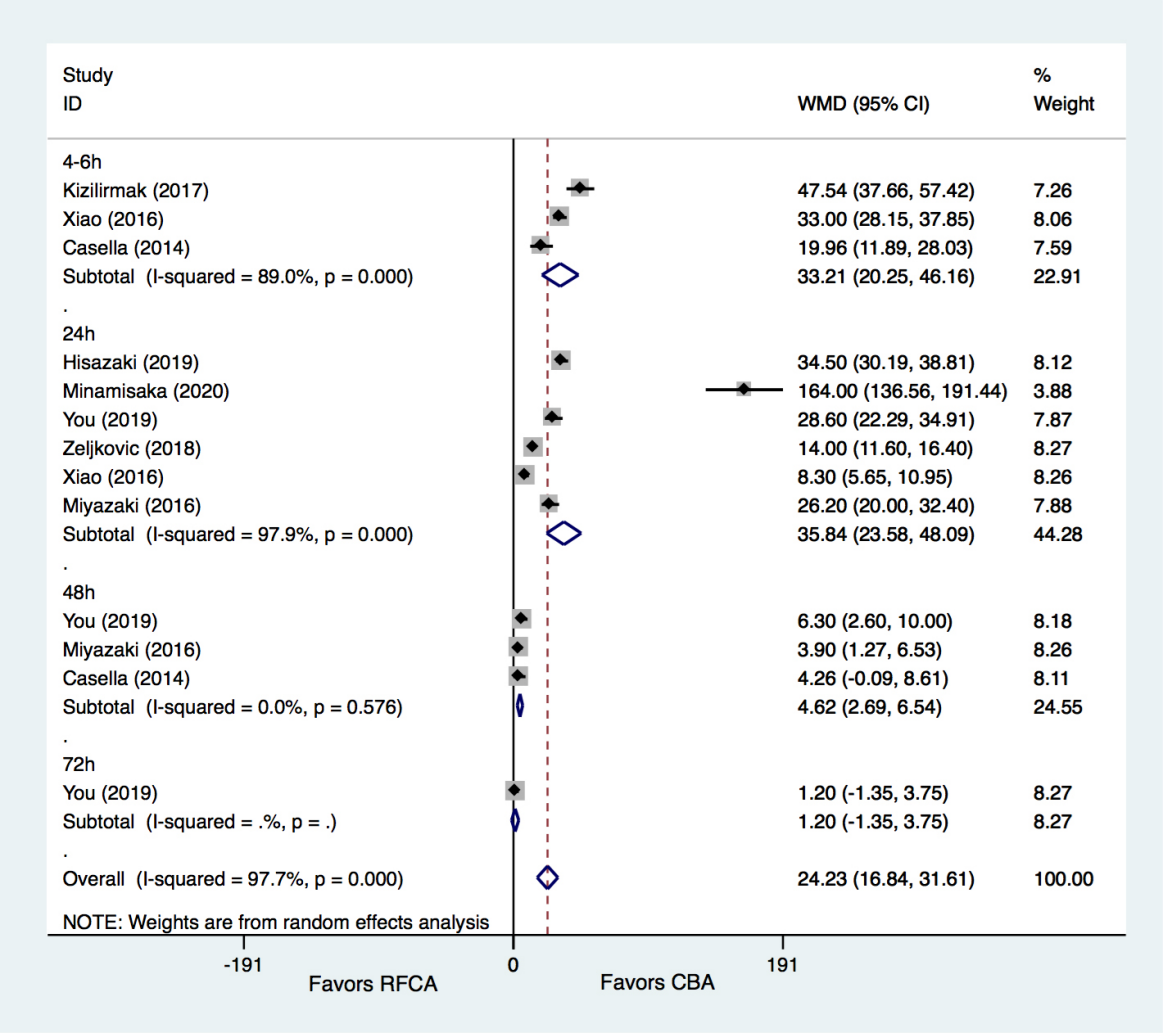
**Meta-analysis for the outcome of CK-MB**. RFCA, radiofrequency catheter ablation; CBA, cryoballoon ablation; WMD, weight mean difference.

Of the included trials, 8 studies [14, 16, 24, 25, 26, 31, 32, 33] provided information on CK-MB levels after ablation in the two groups. The results demonstrated that CBA treatment was associated with a significantly greater increase in CK-MB levels than RFCA treatment (WMD = 24.23 U/L, 95% CI 16.84–31.61, *p* = 0.000). Compared to the RFCA group, the CK-MB levels increased greatly at both 4–6 h (WMD = 33.21 U/L, 95% CI 20.25–46.16, *p* = 0.000), 24 h (WMD = 35.84 U/L, 95% CI 23.58–48.09, *p* = 0.000) and 48 h (WMD = 4.62 U/L, 95% CI 2.69–6.54, *p* = 0.000) in the CBA group. The study by You *et al*. [31] showed similar CK-MB levels between the CBA and RFCA groups at 72 h (WMD = 1.2 U/L, 95% CI –1.35–3.75, *p* = 0.357). However, significant heterogeneities were detected for the comparisons of CK-MB levels at the time points of 4–6 h and 24 h, while no significant heterogeneity was seen at 48 h (Fig. 5).

#### 3.2.4 CK Levels

**Fig. 6.**
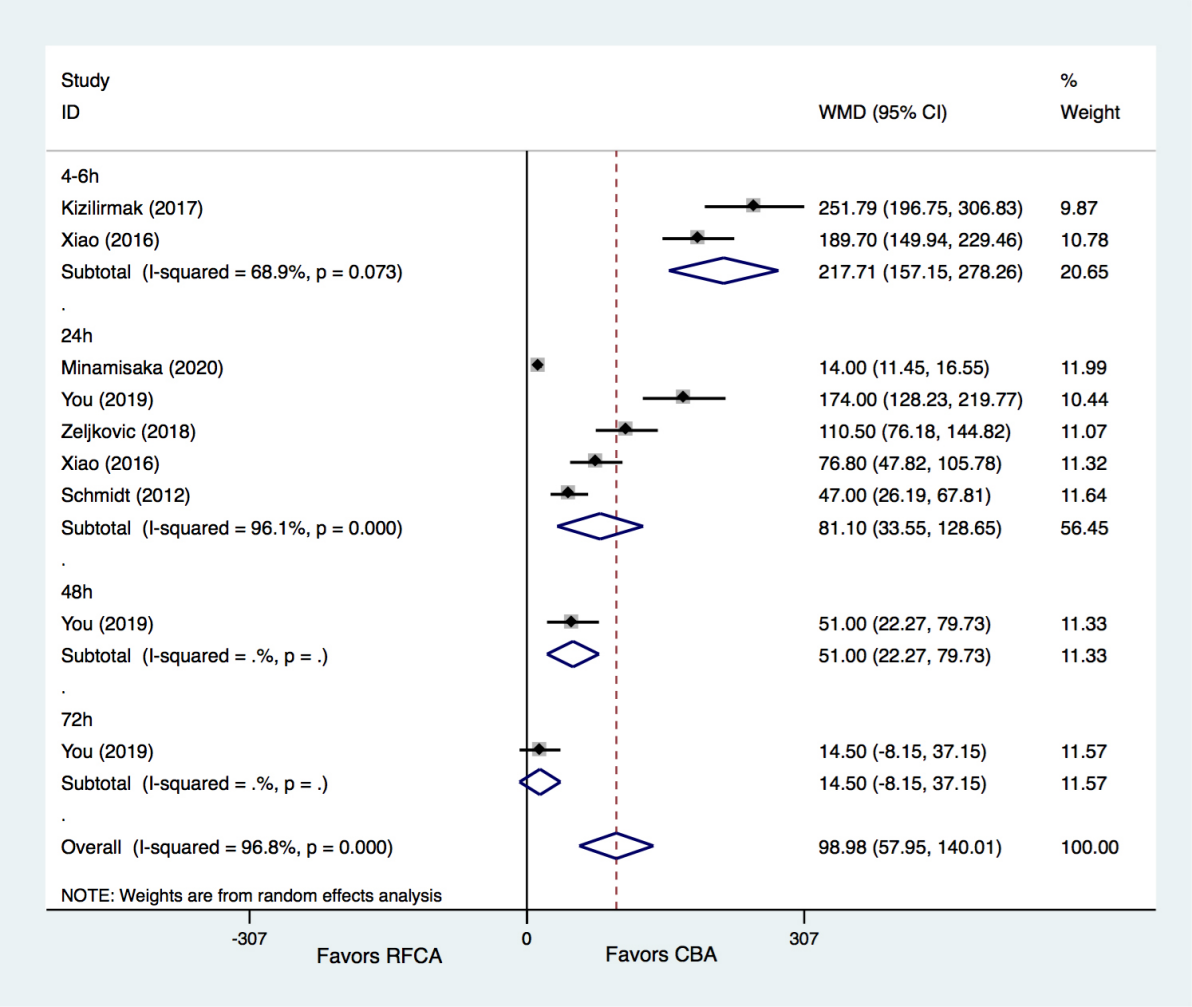
**Meta-analysis for the outcome of CK**. RFCA, radiofrequency catheter ablation; CBA, cryoballoon ablation; WMD, weight mean difference.

Six studies [21, 25, 26, 31, 32, 33] provided information on CK levels after ablation in the two groups. The results demonstrated that CBA treatment was associated with a significantly greater increase in CK levels than RFCA treatment (WMD = 98.98 U/L, 95% CI 57.95–140.01, *p* = 0.000). Compared to the RFCA group, the CK levels increased greatly both at 4–6 h (WMD = 217.71 U/L, 95% CI 157.15–278.26, *p* = 0.000) and 24 h (WMD = 81.10 U/L, 95% CI 33.55–128.65, *p* = 0.001) in the CBA group. The study by You *et al*. [31] showed a greater increase in CK levels at 48 h (WMD = 51.0 U/L, 95% CI 22.27–79.73, *p* = 0.001) in the CBA group, while similar CK levels were observed between the CBA and RFCA groups at 72 h (WMD = 14.5 U/L, 95% CI –8.15–37.15, *p* = 0.210). However, moderate to significant heterogeneities were detected for the comparisons of CK levels at different time points (Fig. 6).

#### 3.2.5 CRP Levels

**Fig. 7.**
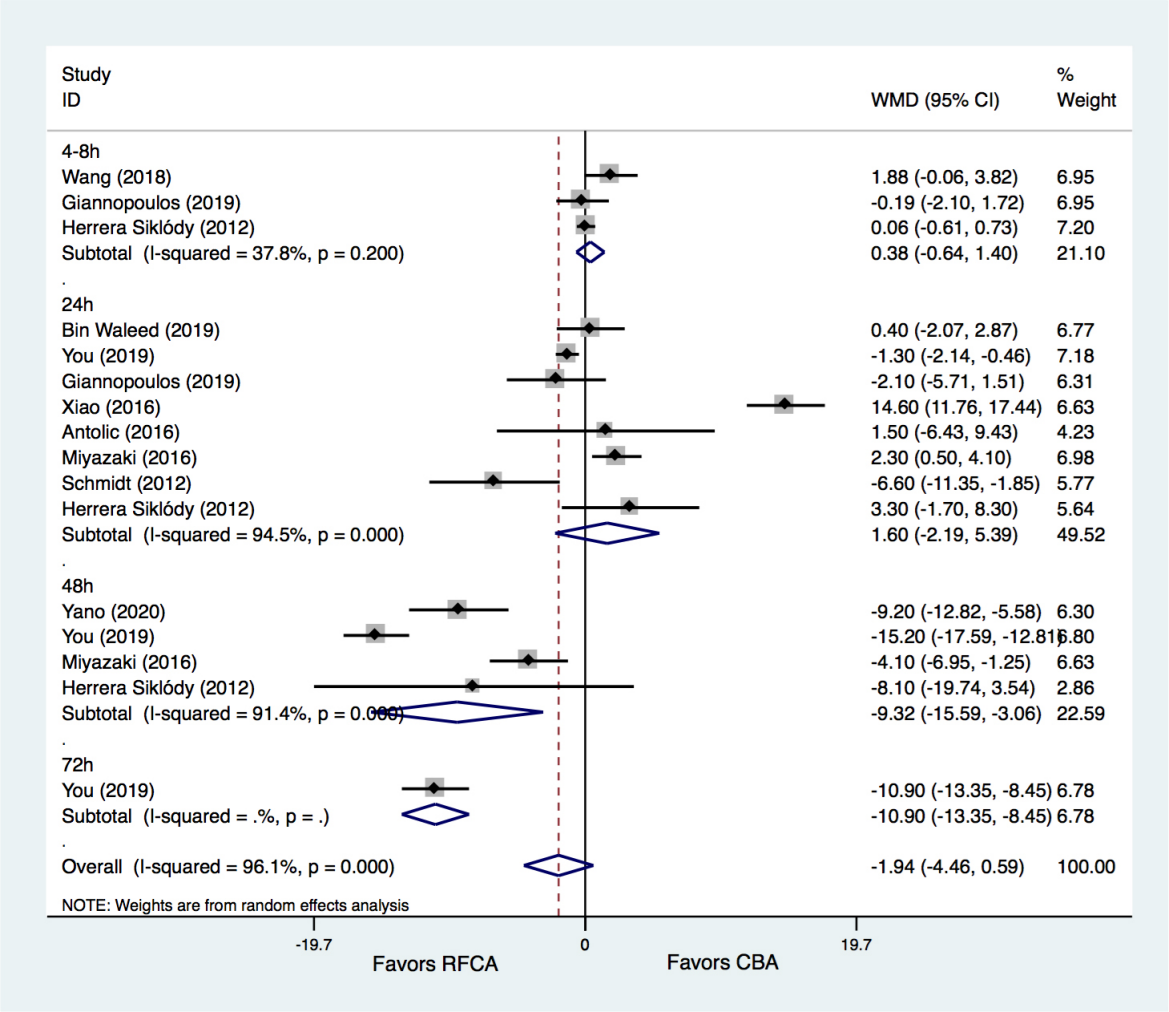
**Meta-analysis for the outcome of CRP**. RFCA, radiofrequency catheter ablation; CBA, cryoballoon ablation; WMD, weight mean difference.

Ten studies [8, 15, 21, 22, 23, 24, 25, 28, 29, 30, 31] provided information on CRP levels after ablation in the two groups. The results demonstrated that the CRP levels were comparable at both 4–8 h (WMD = 0.38 mg/L, 95% CI –0.64–1.40, *p* = 0.464) and 24 h (WMD = 1.60 mg/L, 95% CI –2.19–5.39, *p* = 0.407) between the CBA and RFCA groups. However, significantly greater increases in CRP levels were seen both at 48 h (WMD = –9.32 mg/L, 95% CI –15.59– –3.06, *p* = 0.004) and 72 h (WMD = –10.90 mg/L, 95% CI –13.35– –8.45, *p* = 0.000) in the RFCA group than in the CBA group. Nevertheless, significant heterogeneities were detected for the comparisons of CRP levels at the time points of 24 h and 48 h, while no significant heterogeneity was seen for CRP levels at 4–8 h (Fig. 7).

#### 3.2.6 ERAF and LRAF

**Fig. 8.**
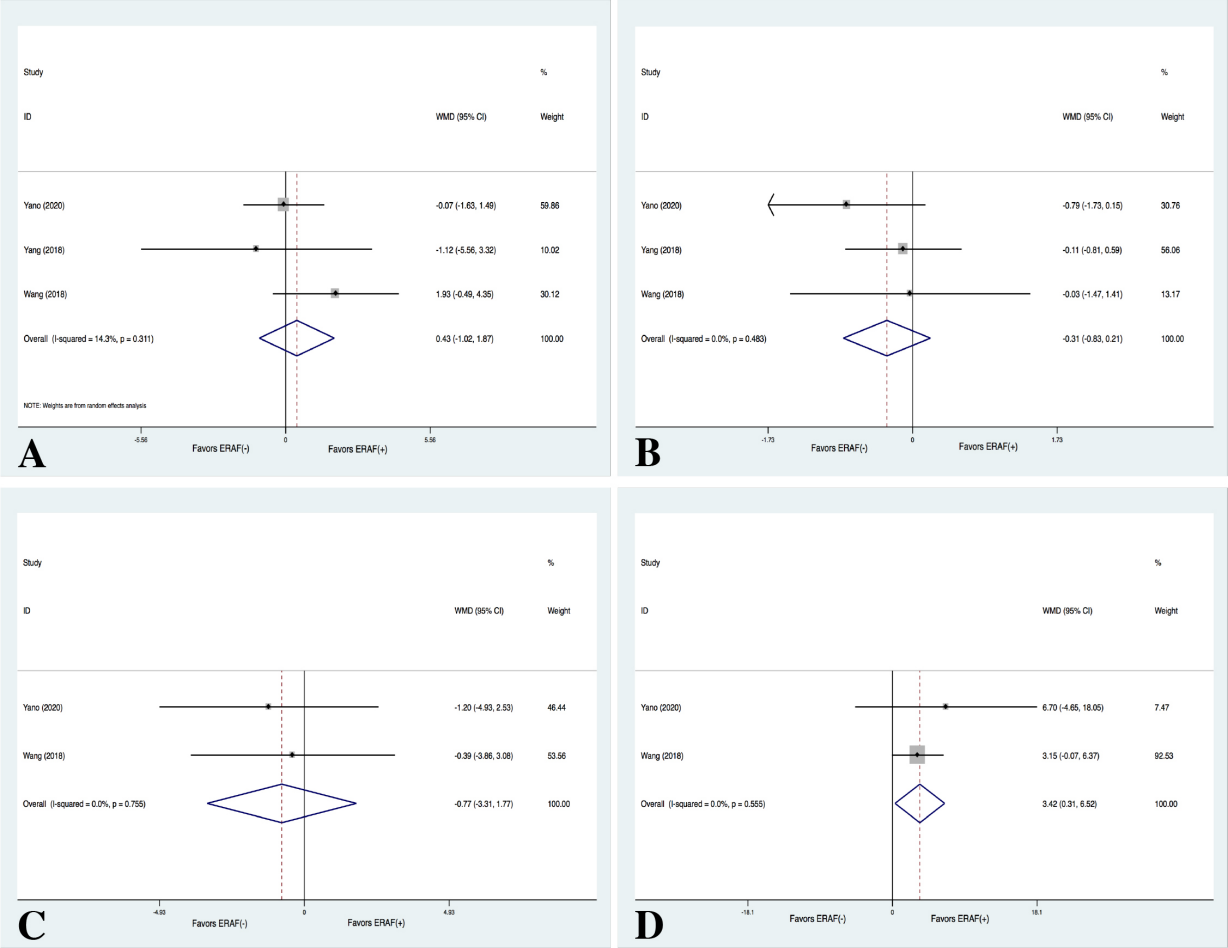
**Meta-analysis for the outcome of TNI and CRP in patients with/without ERAF**. (A) meta-analysis for the outcome of TNI between patients with and without ERAF in the CBA group. (B) Meta-analysis for the outcome of TNI between patients with and without ERAF in the RFCA group. (C) Meta-analysis for the outcome of CRP between patients with and without ERAF in the CBA group. (D) Meta-analysis for the outcome of CRP between patients with and without ERAF in the RFCA group. ERAF, early recurrence of AF; WMD, weight mean difference.

Three studies [8, 27, 28] provided information concerning the effects of myocardial injury and inflammation biomarkers on ERAF. No significant differences were observed between patients with and without ERAF concerning TNI (WMD = 0.427 ug/L, 95% CI –1.016–1.871, *p* = 0.562) and CRP (WMD = –0.766 mg/L, 95% CI –3.306–1.774, *p* = 0.554) after CBA treatment (Fig. 8). Similarly, TNI also did not differ between patients with and without ERAF (WMD = –0.309 ug/L, 95% CI –0.832–0.215, *p* = 0.248) after RFCA treatment. However, the CRP levels were significantly higher in patients with ERAF than in those without ERAF after RFCA treatment (WMD = 3.415 mg/L, 95% CI 0.313–6.517, *p* = 0.031) (Fig. 8). No significant heterogeneity was observed.

**Fig. 9.**
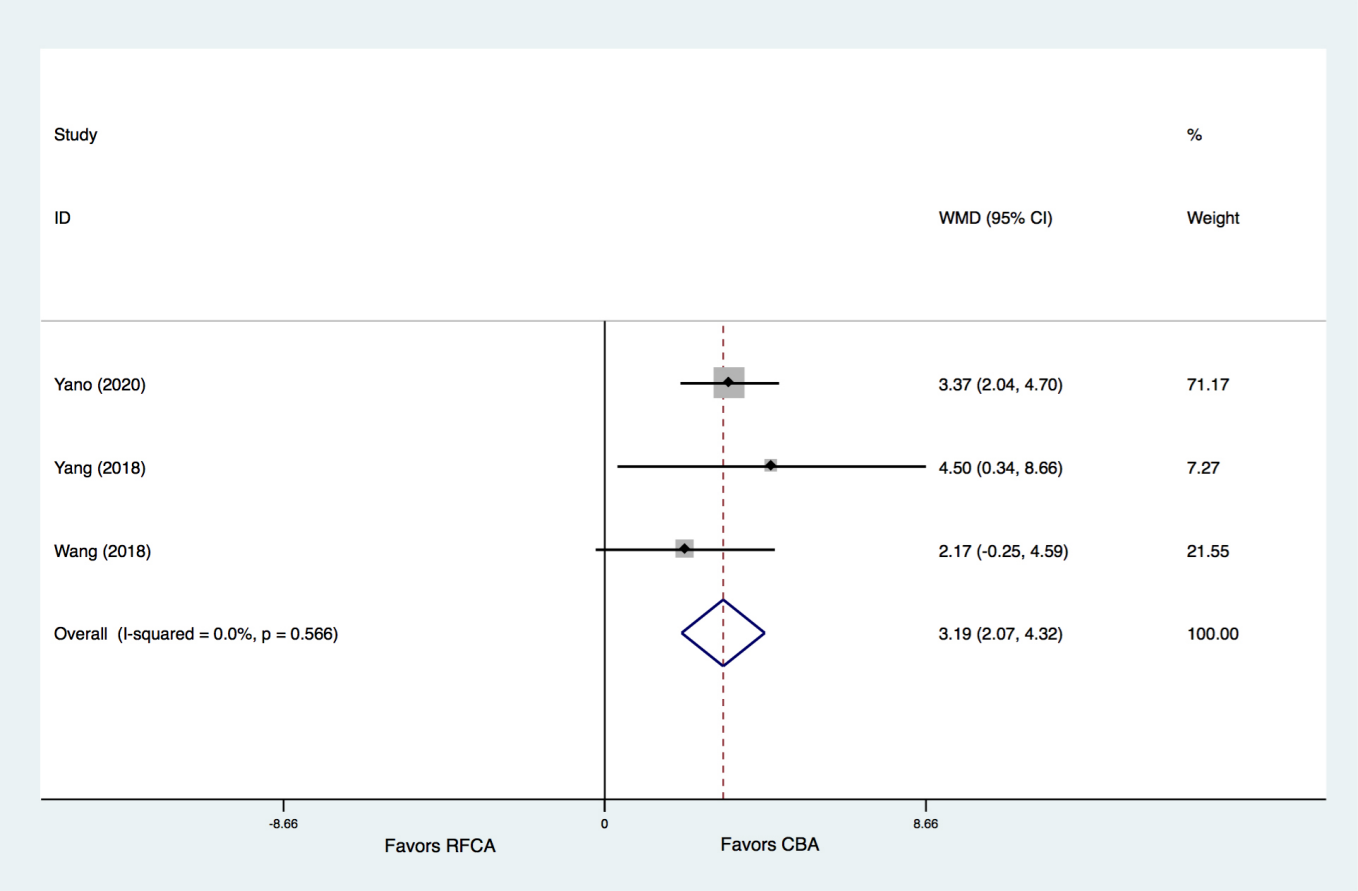
**Meta-analysis for the outcome of TNI in patients with ERAF between CBA and RFCA group**. RFCA, radiofrequency catheter ablation; CBA, cryoballoon ablation; WMD, weight mean difference.

The differences in TNI and CRP levels after the procedure in patients with ERAF between the CBA and RFCA groups were further studied. The results indicated that, the patients with ERAF had significantly higher TNI levels after the procedure in the CBA group than in the RFCA group (WMD = 3.194 ug/L, 95% CI 2.072–4.315, *p* = 0.000) (Fig. 9). No significant heterogeneity was detected. However, the CRP levels after the procedure in patients with ERAF were similar between the two groups (WMD = –7.608 mg/L, 95% CI –21.753–6.537, *p* = 0.292) (**Supplementary Fig. 1**).

Two studies [8, 26] provided information concerning TNI levels after the procedure on LRAF. The results demonstrated that TNI levels did not significantly differ between patients with and without LRAF after the procedure in either the CBA group (WMD = –3.0 ug/L, 95% CI –9.832–3.822, *p* = 0.388) or the RFCA group (WMD = –0.429 ug/L, 95% CI –1.285–0.428, *p* = 0.326) (**Supplementary Figs. 2,3**).

### 3.3 Secondary Endpoints

**Fig. 10.**
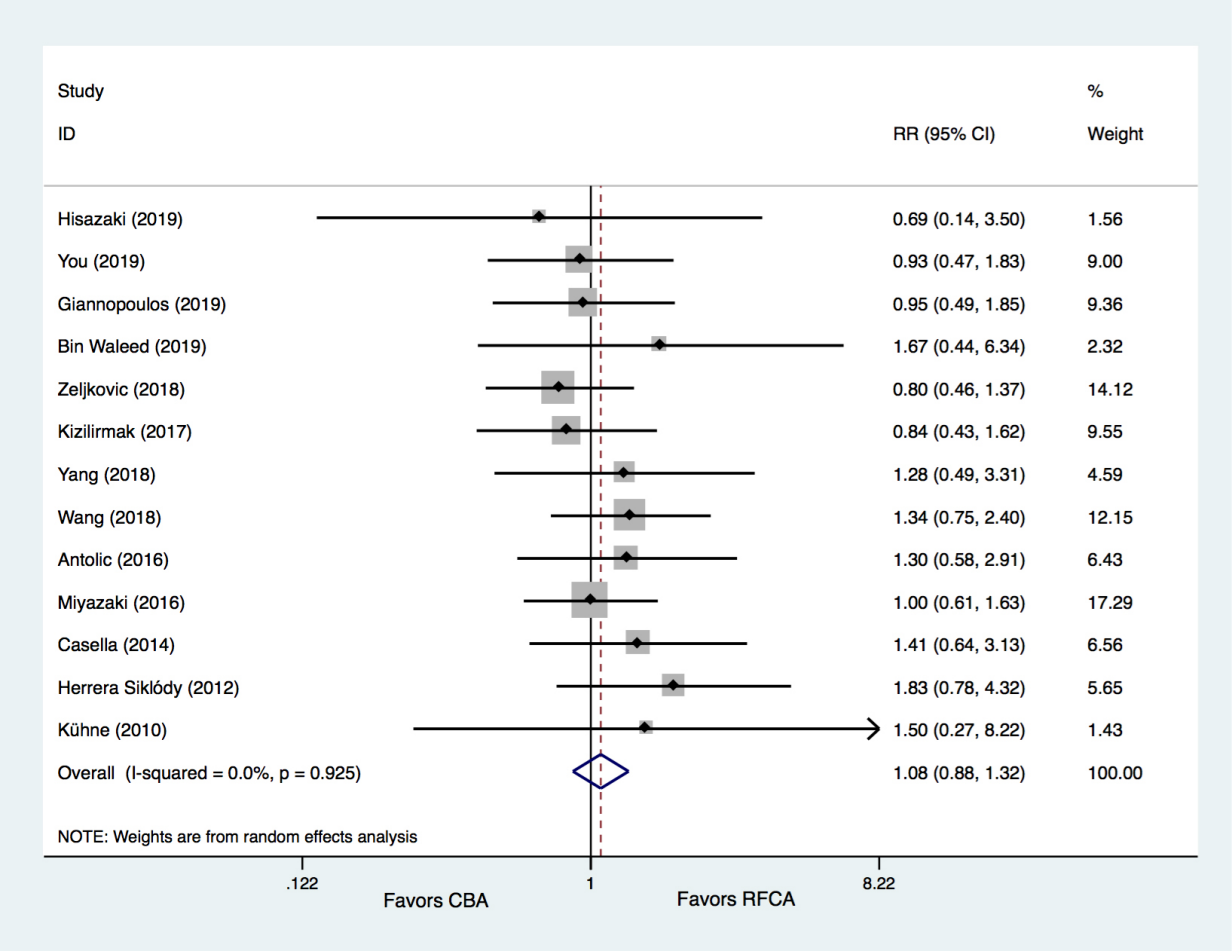
**Meta-analysis for the outcome of freedom form AT**. RFCA, radiofrequency catheter ablation; CBA, cryoballoon ablation; RR, relative risk.

Thirteen studies [14, 15, 16, 17, 23, 24, 26, 27, 28, 29, 30, 31, 32] provided information regarding freedom from AT in both groups. The results demonstrated that freedom from AT did not significantly differ between the CBA and RFCA groups (74.5% vs. 75.2%, RR = 1.08, 95% CI 0.88–1.32, *p* = 0.485). No significant heterogeneity was detected ($I^{2}$ = 0%) (Fig. 10).

Nine studies [8, 15, 16, 17, 21, 22, 25, 26, 29] provided information regarding procedure time in both groups. CBA treatment was found to have a significantly shorter procedure time than RFCA treatment (WMD = –34.57 min, 95% CI –48.85– –20.29 min, *p* = 0.000) (**Supplementary Fig. 4**). However, significant heterogeneity was detected. For the comparison of total ablation time between the CBA and RFCA groups, the results demonstrated that CBA treatment was associated with a significantly shorter ablation time than RFCA treatment (WMD = –517 s, 95% CI –941– –93 min, *p* = 0.017) (**Supplementary Fig. 5**).

## 4. Discussion

The present updated meta-analysis was performed based on 18 clinical studies [8, 14, 15, 16, 17, 21, 22, 23, 24, 25, 26, 27, 28, 29, 30, 31, 32, 33] with a total of 1807 patients. To the best of our knowledge, no previous meta-analysis has comprehensively evaluated the different time-course patterns of myocardial injury and inflammation biomarkers and their impact on recurrence between RFCA and CBA procedures for AF patients. The main findings were as follows: CBA treatment was associated with significantly greater increases in TNI levels, CK-MB levels and CK levels at different time points compared with RFCA treatment; RFCA treatment was associated with significantly higher levels of CRP at 48 h and 72 h; the rate of freedom from AT was comparable between CBA and RFCA treatment; and ERAF was associated with higher postprocedural levels of CRP in the RFCA group.

CA has been established as the primary therapy for the treatment of AF, including RFCA and CBA. Many previous studies, including our preliminary work have investigated the myocardial injury and inflammation biomarkers after RFCA and CBA procedures, and demonstrated that, these biomarkers increased in patients after ablation [8, 9]. However, the results of the published studies were conflicting [14, 15, 17]. The studies by Hisazaki *et al*. [16] and Oswald *et al*. [18] showed significantly higher myocardial injury biomarkers, such as TNT levels in CBA than RFCA, whereas the study by Kuhne *et al*. [17] reported the opposite result. Herrera Siklod́y *et al*. [15] also did not report significant differences in these biomarkers between CBA and RFCA. Evidence regarding to the time-course patterns of these myocardial injury and inflammation biomarkers was also limited.

In the present study, CBA treatment was associated with significantly greater increases in TNI levels, CK-MB levels and CK levels at different time points compared with RFCA treatment. The changes and patterns of these biomarkers indicated that CBA treatment caused much more myocardial injury than RFCA treatment in the acute phase, especially the significantly shorter procedure and ablation time associated with the CBA procedure. In addition, although the overall CRP level did not significantly differ between CBA and RFCA treatment, the CRP levels were significantly higher at 48 h and 72 h after RFCA procedure. The different time-course patterns of the abovementioned biomarker levels may be related to the inherent difference in these two technologies. Previous histological studies have demonstrated that CBA resulted in well-delineated, discrete lesions that preserved the tissue ultrastructure and induced less inflammation [13, 34, 35], and CBA with the second-generation cryoballoon may create much wider injury areas [36]. Conversely, the lesions created by RFCA were characterized by ragged boundaries, with more extensive endothelial cell destruction, leading to increased relapse of endothelial inflammatory cytokines and the activation of platelets and inflammation [37, 38, 39].

The mechanisms of ERAF are incompletely understood. Many studies [17, 26, 27] have investigated the relationships between myocardial injury, inflammation biomarkers and the AF recurrence. In the present study, the proportions of patients who remained free from AT were similar between the CBA group and the RFCA group. Although TNI levels did not significantly differ between patients with and without ERAF or LRAF after the procedure in both the CBA and RFCA groups, patients without ERAF tended to have higher TNI levels, TNT levels, CK levels and CK-MB levels, but have lower CRP levels. In addition, the CRP level was significantly higher in patients with ERAF than in those without ERAF after RFCA.

Inflammation was found to play an important role in the perpetuation of AF and ERAF after RFCA [24, 40], and anti-inflammatory therapy with corticosteroids in the first three days after the ablation procedure was even found to help decrease recurrent arrhythmias [41]. The postprocedure CRP value was found to be a factor correlating with ERAF and LRAF [24]. As CBA treatment was associated with lower levels of CRP, it may result in a lower rate of ERAF, however, the STOP AF trial indicated that ERAF was a common finding, regardless of the application of CBA [42].

Unlike the patterns of myocardial injury biomarkers, such as TNI, the peak of CRP levels came later after the ablation procedure and were especially higher in the RFCA group. The increased CRP levels may represent the postablation inflammatory process, which may be partially related to immediate injury by ablation and systemic abnormalities. Although the inflammatory biomarkers were reported to return to the baseline level approximately 6 months after the procedure [29], patients with systemic abnormalities, such as hypertension and diabetes, may experience a persistent inflammatory process, even in those without ERAF or LRAF, which may promote the process of atrial remodeling and late AF recurrence [43, 44].

Several studies were conducted to investigate the relationships between myocardial injury biomarkers (TNI, TNT, CK and CK-MB) and ERAF after the ablation procedure. The TNT level was found to be associated with advanced atrial arrhythmogenic substrate and a higher incidence of AF recurrence [5, 45]. However, the TNT levels were only evaluated once before the ablation procedure in these studies, and the changes and the levels after the procedure were not analyzed. Other studies showed that smaller increases in the postablation TNT and CK-MB levels were the predictors of ERAF following CBA treatment [44]. A similar result was seen in the study by Kizilirmak *et al*. [26]. The present study also showed that patients without ERAF tended to have higher postprocedural levels of TNI, TNT and CK-MB.

The reduced levels of myocardial injury biomarkers release may be associated with lower lesion dimensions and higher reconnection rates after PVI, and thus lead to worse efficacy of the procedure and clinical outcome, such as an increased ERAF rate [46]. However, the results remain controversial, as the studies by Lim *et al*. [47] and Casella *et al*. [14] showed no significant relationship between postprocedural injury biomarkers and ERAF for a follow-up period of up to 12 months. Notably, nearly half (35–71%) of patients may experience ERAF in a 3-month follow-up [45, 48, 49], but the recurrent episodes in most patients were asymptomatic; therefore, the regular and long-term monitoring of AF is needed after ablation to evaluate the correlations between higher myocardial injury, inflammation biomarkers and clinical outcome [24]. The present study explored the potential mechanisms of ERAF after CBA and RFCA treatment, which may also provide valuable clues for prognostic evaluation and therapeutic decision-making for AF patients. Further large-scale studies with longer follow-up durations are still warranted to comprehensively evaluate the differences in postablation myocardial injury and inflammation biomarkers between CBA and RFCA and to assess their clinical value in predicting AF recurrence.

Certain limitations of this study need to be acknowledged. First, the number of trials included and the sample size were relatively small, especially for the analyses of the impacts of biomarkers on clinical outcomes. Second, both evidence from RCTs and non-RCTs were included, and considerable heterogeneity was detected; thus, the findings should be interpreted with caution. In addition, mixed populations of AF (90.4% PAF vs. 9.6% PerAF) were included, whereas data provided by the included trials limited performing further subgroup analyses. Finally, the follow-up durations were abbreviated, which may be insufficient to evaluate AF recurrence.

## 5. Conclusions

The time-course patterns of postablation myocardial injury and inflammation biomarkers are different between RFCA and CBA procedures. CBA treatment was associated with significantly higher myocardial injury biomarkers at early timepoints, including TNI, CK and CK-MB, while RFCA treatment was associated with higher CRP levels at 48 h and 72 h. CBA and RFCA treatments resulted in comparable rates of freedom from AT, whereas the procedure time and ablation time were significantly shorter in the CBA group than in the RFCA group. TNI levels did not significantly differ between patients with and without ERAF/LRAF in either the CBA or RFCA group. However, patients without ERAF tended to have higher TNI levels after the procedure in both groups, and the CRP levels were significantly higher in patients with ERAF than in those without after RFCA treatment. TNI levels were higher in patients with ERAF after procedure in the CBA group than in the RFCA group. The reduced postprocedural elevation of myocardial injury biomarkers, including TNI levels, and the significantly increased CRP levels, may be predictive factors for ERAF. Further large-scale studies with longer follow-up durations are needed to provide up-to-date evidence.

## Supplementary Material

Supplementary material associated with this article can be found, in the online version, at https://doi.org/10.31083/j.rcm2312397.
